# Management of a Mandibular Odontogenic Keratocyst with Enucleation, Piezotome-Assisted Peripheral Ostectomy, and Platelet-Rich Fibrin—A Case Report

**DOI:** 10.3390/dj13110536

**Published:** 2025-11-14

**Authors:** Ehab Abdelfadil, Maha Alsharif, Alla T. Alsharif, Samah Mourad

**Affiliations:** 1 Department of Oral and Maxillofacial Surgery, Faculty of Dentistry, Mansoura University, Mansoura 35516, Egypt; ehabident@mans.edu.eg; 2 Department of Oral Diagnostic Sciences, Faculty of Dentistry, King Abdulaziz University, Jeddah 21589, Saudi Arabia; 3 Department of Preventive Dental Sciences, College of Dentistry, Taibah University, Al-Madinah Al-Munawwarah 42353, Saudi Arabia; ashref@taibahu.edu.sa; 4 Department of Oral & Maxillofacial Diagnostic Sciences, College of Dentistry, Taibah University, Al-Madinah Al-Munawwarah 42353, Saudi Arabia; smourad@taibahu.edu.sa

**Keywords:** odontogenic keratocyst, mandible, piezosurgery, platelet-rich fibrin, bone regeneration

## Abstract

Background/Objectives: Odontogenic keratocyst (OKC) is a benign yet locally aggressive intraosseous lesion with a high recurrence rate, posing significant challenges for clinical management. This present case report describes a combined surgical management approach for an OKC, involving enucleation with piezotome-assisted peripheral ostectomy, followed by adjunctive application of platelet-rich fibrin (PRF) to the osseous defect. Methods: A 54-year-old patient presented with a mandibular unilocular radiolucency that was diagnosed histopathologically as an OKC. The lesion was managed using a combined surgical approach involving enucleation and peripheral ostectomy with a piezotome. To optimize healing, PRF was applied to the bone defect. Results: At 18- and 36-month follow-up, the patient demonstrated complete postoperative bone remodeling of the cystic bone defect, with no evidence of recurrence. Conclusions: This case highlights the low morbidity associated with enucleation and piezotome-assisted peripheral ostectomy, which may be preferable to other aggressive OKC treatment modalities in selected cases. Additionally, PRF use was associated with favorable postoperative healing, with minimal pain, swelling, and inflammation. Radiographic stability at 36 months supports the feasibility of this approach; however, larger studies are needed to compare recurrence risk with other interventions.

## 1. Introduction

Odontogenic keratocyst (OKC) is a benign but locally aggressive intraosseous jaw lesion first described by Philipsen in 1956 [1]. It accounts for 2–21.8% jaw cysts [2,3], and has a propensity to spread rapidly and invade surrounding tissues [3,4]. In 2005, the World Health Organization (WHO) classified it as a keratocystic odontogenic tumor (KCOT) because of its aggressive behavior, high recurrence rate (0–62%), presence of daughter (satellite) cells, mutations in tumor suppressor genes, and association with Gorlin–Goltz syndrome. However, in the fourth edition of Current Diagnosis and Treatment, the WHO reclassified OKC as an odontogenic developmental cyst due to insufficient evidence supporting a neoplastic origin or distinct biological behavior [5,6].

OKC appears to be more prevalent in men [7], although some studies report no gender differences [8]. More lesions are detected in the second to fourth decades of life, with about 60% diagnosed between the ages of 10 and 40 years. Approximately 60–80% arise in the posterior mandible and the lower ascending ramus [9]. Localized, often asymptomatic swelling is the most common presentation. Spontaneous drainage into the oral cavity and variable tooth mobility are also reported. Less common clinical symptoms include nasal airway obstruction, paresthesia, and resorption of roots of associated teeth. The majority of cases are asymptomatic and discovered incidentally on routine radiographic examination [10].

Initial dental imaging typically includes panoramic and periapical radiographs. Advanced modalities, such as three-dimensional (3D) computed tomography and/or magnetic resonance imaging, are often required—particularly for large multilocular lesions—to identify the extent of soft- and hard-tissue involvement. Radiographically, OKC typically presents as a well-defined unilocular or multilocular radiolucency with a sclerotic margin. The differential diagnoses include lateral periodontal cyst, traumatic bone cyst, periapical radicular cyst (especially when located in the anterior maxillary area), dentigerous cyst, central giant cell granuloma, ameloblastoma, adenomatoid odontogenic tumor, and other bone tumors. Even when aspiration is performed after clinical and radiographic assessment, histopathological examination is mandatory to establish a definitive diagnosis [11].

Multiple treatment approaches aim to improve patient outcomes and reduce the risk of recurrence and potential morbidity. Broadly, they are categorized as conservative—simple enucleation (with or without curettage), decompression, or marsupialization—and non-conservative (aggressive)—enucleation with peripheral ostectomy, enucleation with Carnoy’s solution, cryotherapy, and surgical resection [2,3]. Selection should consider both patient- and lesion-related factors. Patient factors include age, syndrome association, and medical comorbidities; lesion factors include size, location, extent, cortical perforation, and involvement of surrounding soft tissue and adjacent structures. Retreatment can be tailored to the pattern of recurrence. More aggressive options may be warranted, but recurrence risk must be balanced against functional outcome and possible morbidity [10,12]

Piezosurgery is a relatively novel, precise, and safe technique for ostectomy in oral and maxillofacial surgery [13]. Although relatively few reports focus specifically on jaw cysts and tumors, the modality is well established. A growing body of clinical and experimental evidence supports the effectiveness of piezoelectric bone cutting for ostectomy-type procedures and peripheral bone removal, with favorable outcomes compared with conventional rotary instruments, as it selectively cuts mineralized tissue without damaging soft tissue [13,14,15]. In a randomized trial of mandibular third-molar surgery, piezosurgery for ostectomy was associated with significantly less postoperative inflammation (pain, swelling, trismus) than bur-based ostectomy, but with slightly longer operative time [13]. Ultrasonic instruments also enable precise bone cutting with less operating pressure and can be used safely near vital structures [16].

After cyst enucleation, the residual bony cavity poses a clinical challenge, and promoting bone regeneration remains a key concern [17]. Some reports have supported the use of filler materials to enhance healing, whereas others favor spontaneous bone regeneration without grafting. Autologous platelet concentrations, especially platelet-rich fibrin (PRF), have shown favorable outcomes in promoting postoperative healing and enhancing tissue regeneration and repair after cyst removal [17,18,19].

There is no universally accepted surgical protocol for OKC. Conservative approaches are not necessarily associated with higher recurrence [4]. Further studies are recommended to compare surgical modalities and clarify their impact on recurrence [4,20,21]. This case report describes the outcomes of managing OKC with enucleation and piezotome-assisted peripheral ostectomy, followed by PRF placement in the resultant bony cavity.

## 2. Case Presentation

### 2.1. History and Examination

A 54-year-old man was referred to the oral and maxillofacial department of an outpatient dental center with a chief complaint of spontaneous dull pain in the lower left posterior mandible in the premolar areas that started a few weeks ago. The patient also reported mild tenderness on palpation in the overlying cheek area. No neurosensory disturbances associated with the left mental nerve were described. There were no significant findings from the patient’s past medical and family histories.

**Figure 1:**
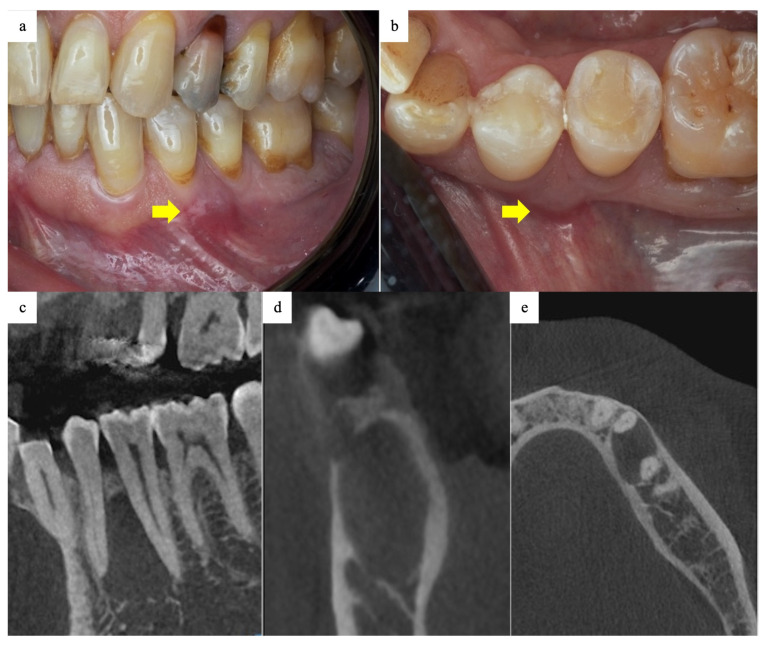
(**a**,**b**) Preoperative clinical photographs showing a mild swelling between teeth 35 and 36 (arrow) with normal covering mucosa. (**c**–**e**) Preoperative CBCT showing a well-defined radiolucent area related to teeth 35 and 36.

Extraoral examination revealed facial symmetry with no lymphadenopathy or draining fistula. The patient had a normal mouth opening without any temporomandibular joint disorders. Intraoral examination showed mild intraoral swelling located between teeth 34 and 35, with normal covering mucosa (Figure 1a,b). The first molar had superficial dental caries. Mild generalized gingival recession was also observed. The mandibular left posterior teeth showed unremarkable mobility, with mild tenderness on percussion. Teeth 35 and 35 were vital, with a normal response.

Aspiration was performed through bone fenestration detected by cone beam computed tomography (CBCT), and a thick, yellowish keratinous fluid was revealed. Intraoral periapical film and full mouth orthopantomogram (OPG) showed a well-defined radiolucency related to the roots of the mandibular left premolar teeth. A CBCT scan revealed a radiolucent lesion with dimensions of 1.5 cm × 1.7 cm × 0.9 cm. The lesion was in close proximity to the mental nerve, with no direct involvement (Figure 1c–e).

The differential diagnosis of the lesion was discussed with the patient, along with different treatment modalities and risk factors. The patient refused to extract the teeth and requested a more conservative initial treatment and follow-up. A well-formulated treatment plan was created, consisting of presurgical endodontic treatment of teeth 34 and 35, followed by enucleation of the lesion, curettage, and peripheral ostectomy using piezotome-assisted peripheral ostectomy.

### 2.2. Surgical Phase

**Figure 2:**
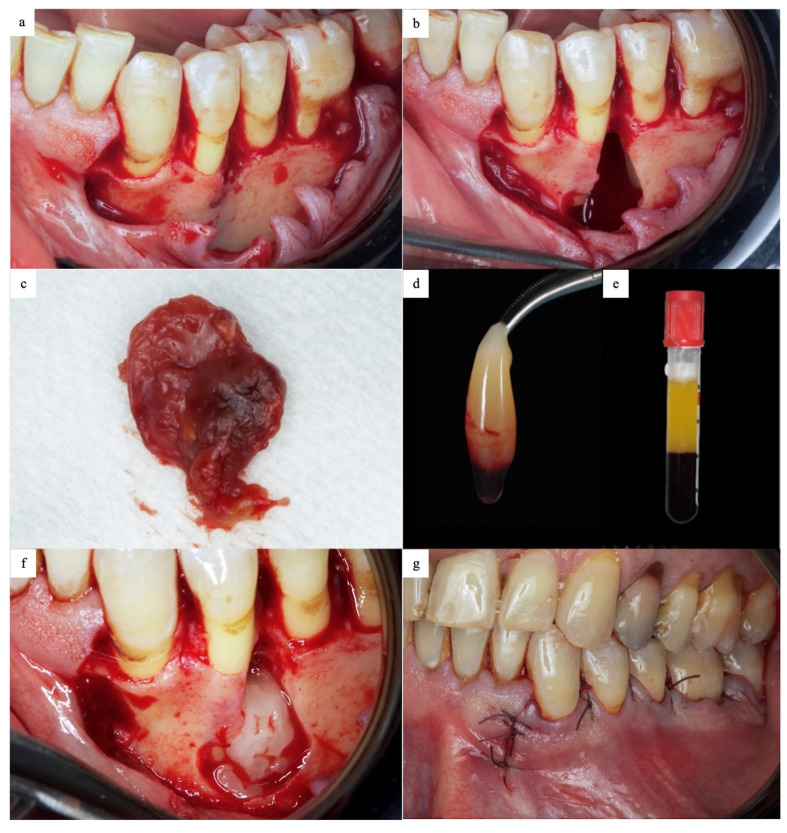
(**a**) Clinical photograph after flap reflection showing bone fenestration between teeth 34 and 35. (**b**) Clinical photograph showing the bone cavity after cyst removal. (**c**) Curettage and the enucleated lesion. (**d**) PRF after cellular separation and PRF formation following centrifugation. (**e**) The PRF clot was separated from the upper part of the RBC layer. (**f**) Clinical photograph showing packing the cavity with PRF. (**g**) Flap repositioning and suturing.

Under local anesthesia, a full-thickness flap consisting of a sulcular incision and one oblique incision mesial to the canine was utilized. The flap was incised and reflected to expose the alveolar bone. After flap reflection, a small cortical perforation was seen at the crest between teeth 34 and 35 (Figure 2a). The perforation was enlarged using piezosurgery (Solo LED, Acteon, Piezotome, France) to create a bony window to access the lesion. The lesion was enucleated using a surgical curette, and apicectomy of teeth 34 and 35 was performed (Figure 2b,c). Piezosurgery was then used to conduct peripheral ostectomy using a large diamond-coated tip. Subsequently, the bone edges were smoothened, and the cavity was irrigated copiously with a saline solution. The freshly prepared PRF (Figure 2d,e) was then packed in the cystic cavity, and the flap was repositioned and sutured using 4/0 polyglycolic acid sutures (Figure 2f,g).

The patient was given verbal and written postoperative instructions, which included intermittent extraoral application of cold packs, avoidance of hot food and drinks on the first day, and application of warm compresses starting on the second day. Postoperative medications included 875 mg of amoxicillin combined with 125 mg of clavulanic acid (Augmentin 1 g, SmithKline Beecham Pharmaceutical Co., Brentford, UK) to be taken twice daily for seven days after surgery and 50 mg of diclofenac potassium (Cataflam 50 mg tablets, Novartis Pharma AG, Basel, Switzerland), a non-steroidal anti-inflammatory drug, to be taken three times daily for 7–10 days. The patient was also instructed to use a chlorhexidine gluconate mouthwash (Kin Gingival Complex, Laboratorios KIN S.A., Barcelona, Spain) for 10 days. The sutures were removed 10 days after the procedure.

### 2.3. Postoperative Evaluation

The patient was seen on the second and seventh days postoperatively for wound evaluation and on the tenth day for suture removal. The patient was instructed to report to the surgeon immediately in case of severe pain or any other emergency.

### 2.4. Histology

**Figure 3:**
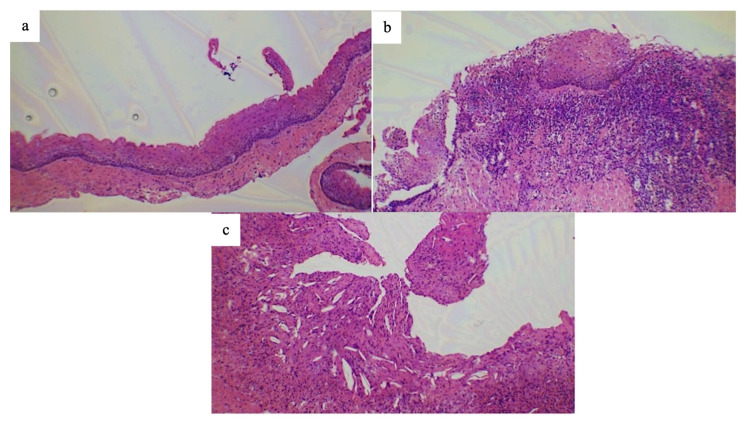
Histologic analysis by Hematoxylin–Eosin staining revealed (**a**) a cystic lesion lined by parakeratinized epithelium with a corrugated surface and a hyperchromatic, palisaded basal cell layer (×10 magnified photomicrograph). (**b**) Subepithelial connective tissue showing mixed inflammatory cell infiltrate. (**c**) Foci of cholesterol cleft formation within the cystic wall (×10 magnified photomicrograph).

Microscopic examination revealed a cystic lesion lined by parakeratinized stratified squamous epithelium with a corrugated surface. The epithelium measured approximately 6–8 cell layers in thickness, with a hyperchromatic, palisaded basal layer and a flat epithelial–connective tissue interface. In some areas, the subepithelial connective tissue showed a prominent mixed inflammatory infiltrate composed of lymphocytes, plasma cells, and neutrophils, with foci of cholesterol clefts. There was no microscopic evidence of ameloblastic proliferation, cytologic atypia, or malignancy. No histological predictors of aggressive behavior, such as satellite (daughter) cysts, were identified (Figure 3a,c). Based on these findings, the lesion was diagnosed as a secondary inflamed OKC.

### 2.5. Follow-Up and Outcomes

The patient complied with postoperative instructions and medication. Periodic clinical and radiographic monitoring with OPG and CBCT was undertaken. Recurrence was considered present if any of the following occurred: clinically, new or persistent swelling, pain, infection or drainage, altered sensation in the mental nerve distribution, or functional limitation; radiographically, (1) a new or enlarging radiolucency at or adjacent to the original site on serial OPG/CBCT; (2) cortical thinning, expansion, or perforation; (3) loss of internal trabeculation; or (4) failure of defect reduction within 6 months postoperatively [22,23,24]. Histologic confirmation was planned if clinical and/or radiographic suspicion of recurrence arose.

**Figure 4:**
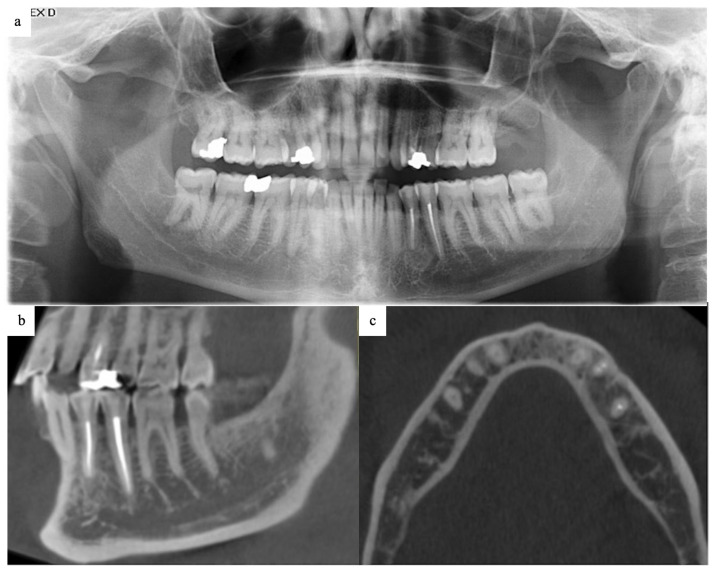
(**a**) Full-mouth OPG taken 18 months after the surgery, showing normal bone regeneration at the site of the enucleated lesion. (**b**,**c**) CBCT taken 36 months after the surgery, showing uneventful bone healing of the cystic bone defect without evidence of lesion recurrence.

At 9 months, OPG and CBCT demonstrated bone remodeling with no evidence of recurrence. An 18-month follow-up showed normal bone regeneration on OPG imaging (Figure 4a). At 3 years postoperatively, the patient reported no altered sensation; light-touch testing over the mental nerve distribution was normal, occlusion was unchanged, and analgesic use had been limited to the first postoperative week. Uneventful healing with complete remodeling of the cystic bone defect was observed (Figure 4b,c). Given the potential late recurrences, surveillance for ≥10 years has been incorporated into the patient’s long-term follow-up plan.

## 3. Discussions

The reclassification of OKC from a benign tumor to a developmental odontogenic cyst reflects evolving evidence regarding its biological behavior and morphogenesis. Key findings include PTCH1 mutations that are not specific to OKC and regression following marsupialization/decompression. In addition, non-neoplastic spontaneous transformation of the cystic lining to normal oral epithelium supports the updated nomenclature and informs treatment selection [4]. Management options range from conservative (marsupialization or decompression) to aggressive (bone resection). Choice of modality depends primarily on the patient factors (age, ability to adhere to follow-up) and lesion characteristics (location, size, proximity to vital structures, and history of recurrence) [25]. There is no consensus on a single best approach. Given the lesion’s benign nature, the optimal strategy is the one that minimizes morbidity, preserves anatomy and function, and reduces postoperative complications [4,20].

In this case, enucleation followed by peripheral ostectomy was selected, with adjunctive PRF to enhance bone healing. Following enucleation, the cystic capsule was removed while maintaining mandibular continuity and preserving the teeth and mental nerve. This aligns with studies favoring enucleation when critical anatomy is not compromised and esthetic or functional risks are low [4,19]. Peripheral ostectomy was added to improve the removal of OKC epithelial remnants and reduce recurrence risk and postoperative complications [20,26]. The presence of an intact fibrous capsule during curettage further supports the decision to perform peripheral ostectomy in this case [26].

Piezosurgery appears to improve outcomes when peripheral ostectomy is incorporated into the treatment protocol for OKC [27]. Its effectiveness for bone removal is supported across a range of oral surgical procedures, including sinus lift, block harvesting, implant site preparation, and excision of large osteomas. Randomized trials and a recent meta-analysis comparing piezosurgery with rotary instruments for enucleation of jaw cysts/tumors report better hemostasis and visibility, fewer early postoperative complications, and no increase in soft-tissue injury or paresthesia, though with longer operative time; long-term recurrence is not higher than with conventional bur [14,28,29,30,31]. In a retrospective case–control study by Troiano et al., enucleation with piezo-assisted peripheral ostectomy for solid/multicystic ameloblastoma was associated with a lower 5-year relapse rate than conventional drilling, supporting clinical effectiveness in practice [32].

In the present case, a lesion adjunct to the mental nerve in the left mandibular premolars was completely enucleated without temporary or permanent paresthesia during three years of follow-up. Experimental and clinical data suggest that piezosurgical instruments better preserve osteocyte vitality than rotary tools and cause less bone necrosis, potentially prompting earlier bone healing and reducing intraoperative/postoperative bleeding. Although enucleation with peripheral ostectomy is considered more aggressive than decompression or marsupialization, it has been associated with lower surgical morbidity than resection and then enucleation with Carnoy’s solution in some series [3,27,33].

Complete removal of the cystic lining is critical to minimizing recurrence. Reported recurrence after enucleation with peripheral ostectomy ranges from 17.4 to 18.2%, which is higher than rates after resection (1.85–2.2%) and after application of Carnoy’s solution (4.8–5.3%) [3,24]. Resection, however, carries substantial functional and esthetic morbidity and is generally reserved for large, recurrent, or anatomically challenging lesions [3,34]. Carnoy’s solution chemically cauterizes residual epithelial remnants to a depth of approximately 1.5 mm [20] but has reported adverse effects, such as wound dehiscence, infection, and transient neurotoxicity/paresthesia, particularly when applied near the inferior alveolar nerve [35].

A 2018 retrospective series by Karaca et al. reported a 14.8% recurrence rate among 27 patients managed with enucleation with peripheral ostectomy over a median five-year follow-up, concluding that the approach is effective and safe with low morbidity; careful preoperative imaging and meticulous surgery by experienced clinicians may further reduce recurrence [3]. The influence of lesion localization on recurrence remains controversial: Kaczmarzyk et al. (2012) found higher recurrence in the posterior mandible [27], whereas other studies found no significant association with size and radiographic pattern [36,37]. Recurrence likely arises via (1) incomplete removal of the original cyst, (2) new cyst formation from residual satellite (daughter) cysts, or (3) development of a new keratocyst adjacent to the original site that can be mistaken for recurrence [38,39].

Variation in reported recurrence rates likely reflects both biological and methodological heterogeneity: follow-up duration and imaging intervals, inconsistent definitions of recurrence, and mixed case characteristics (e.g., size > 3 cm, multilocularity, posterior mandible/ramus location, tooth association, cortical perforation, nevoid basal cell carcinoma syndrome, histologic subtype). Treatment factors—margin control, adjuncts (peripheral ostectomy, chemical cautery, cryotherapy), and surgeon experience—also influence outcomes. Our strategy combined enucleation with piezo-assisted peripheral ostectomy to optimize epithelial debridement while minimizing the mental nerve risk. For high-risk or refractory disease (multilocular, >3 cm, persistent after decompression, cortical perforation), more aggressive measures, including resection with removal of the involved mucosa/soft tissue, may be needed [24].

Peripheral ostectomy after enucleation can be performed with rotary burs, often aided by vital stains (e.g., methylene blue/crystal violet) to highlight residual epithelial remnants; typically, 1–2 mm of bone is removed until punctate bleeding is observed, although high-quality comparative trials are limited [40]. Carnoy’s solution historically lowers recurrence when applied to cavity wall (1–3–5 min protocols), but neurotoxicity concerns and regulatory constraints on chloroform have led to modified formulations with mixed results [41]. Cryotherapy with liquid nitrogen (spray/probe) devitalizes epithelial remnants to a depth of ~1.5 mm and, when combined with enucleation, tends to reduce recurrence versus enucleation alone, but temporarily weakens bone with fracture risk at around eight weeks [24]. Network and pairwise meta-analyses increasingly highlight topical 5-FU as a targeted adjunct with less postoperative morbidity than conventional approaches, although broader validation is needed [8].

Among commonly used modalities, enucleation is efficient, often completed in a single session, and relies on centripetal bone healing of the residual cavity [42]. Ongoing bone remodeling permits spontaneous healing without adjunct graft in many cases [43]. In this case, PRF—a second-generation platelet concentrate—was placed in the cavity after enucleation and peripheral ostectomy. Evidence suggests PRF enhances bone healing and soft-tissue regeneration after cyst enucleation and may reduce bone resorption compared with ungrafted healing [44,45], thereby decreasing discomfort related to delayed healing and potentially recurrence risk [19,46]. Proposed mechanisms include the gradual release of cytokines and growth factors, which stimulate collagen synthesis and osteogenesis [47]

A systematic review assessing PRF after odontogenic cyst enucleation reported the fastest bone fill and lowest pain scores in PRF-treated patients versus PRF and graft, graft only, or spontaneous healing, with no significant adverse effects [46]. Additional reports attribute analgesia to reduced inflammation, and note reduced bleeding, rapid vascularization, and accelerated soft tissue and bone healing, consistent with the role of early hemostasis of platelets and fibrin clot formation. PRF may also support antimicrobial defense via chemotactic cytokines and immunomodulatory signals (e.g., interleukin-4). Consistent with these findings, this secondarily inflamed OKC showed favorable postoperative healing with minimal pain, swelling, or inflammation during follow-up [19,46,47]. Surgery remains the mainstay for odontogenic cysts [6]. Our case report suggests that enucleation with piezotome peripheral ostectomy can achieve low morbidity and may be preferable to more aggressive modalities in selected cases. PRF is a simple, cost-effective adjunct with the promising potential to improve soft-tissue healing and accelerate bone regeneration. Despite the ongoing risk of recurrence, extended postoperative evaluation is essential to ensure durable control and early detection.

Given that this is a single case, results should be interpreted cautiously. Although the three-year follow-up showed favorable healing, surveillance for ≥10 years is advisable because of late recurrence. Future studies should incorporate standardized patient-reported outcomes and a larger cohort to compare recurrence across interventions.

## 4. Conclusions

Management of OKC remains challenging due to its recurrence potential and locally aggressive behavior. In this case, accurate diagnosis, including a thorough history and examination supported by preoperative 3D imaging and biopsy, enables tailored management and improves outcome. Treatment decisions should be individualized based on patient factors and histopathology. This combination of OKC enucleation with piezotome-assisted peripheral ostectomy and PRF placement yielded favorable healing with minimal morbidity. Because recurrence can occur late, long-term clinical and radiographic follow-up is essential, and the patient’s adherence is critical for early detection of relapse.

## Data Availability

The original contributions presented in this study are included in the article. Further inquiries can be directed to the corresponding author.

## Abbreviations

OKC—odontogenic keratocyst; PRF—platelet-rich fibrin; WHO—World Health Organization; KCOT—keratocystic odontogenic tumor; OPG—orthopantomogram; CBCT—cone–beam computed tomography; RBC—red blood cell; 3D—three-dimensional.
